# One Step In-Situ Synthesis of Zinc Oxide Nanoparticles for Multifunctional Cotton Fabrics

**DOI:** 10.3390/ma14143956

**Published:** 2021-07-15

**Authors:** Asif Javed, Jakub Wiener, Asta Tamulevičienė, Tomas Tamulevičius, Algirdas Lazauskas, Jana Saskova, Simas Račkauskas

**Affiliations:** 1 Department of Material Engineering, Faculty of Textile Engineering, Technical University of Liberec, 46001 Liberec, Czech Republic; jakub.wiener@tul.cz (J.W.); jana.saskova@tul.cz (J.S.); 2 Institute of Materials Science, Kaunas University of Technology, K. Baršausko St. 59, LT-51423 Kaunas, Lithuania; asta.tamuleviciene@ktu.lt (A.T.); tomas.tamulevicius@ktu.lt (T.T.); algirdas.lazauskas@ktu.lt (A.L.); simas.rackauskas@ktu.lt (S.R.); 3 Department of Physics, Kaunas University of Technology, Studentų St. 50, LT-51423 Kaunas, Lithuania

**Keywords:** ZnO, nanomaterials, metal oxide, antibacterial, UV protection, self-cleaning

## Abstract

Zinc oxide nanoparticles (ZnO NPs) have acquired great significance in the textile sector due to their impressive efficiency and multifold utilization, such as antimicrobials, UV protection, photo catalytic activity, and self-cleaning. The aim of this work is in-situ growth of ZnO NPs on 100% cotton fabrics with the one-step hydrothermal method for preparation of multifunctional textile with UV protecting, antibacterial, and photo catalytic properties. Sodium hydroxide (NaOH) and Zinc nitrate hexahydrate [Zn(NO_3_)_2_·6H_2_O] were used as reactants for the growth of zinc oxide on the 100% cotton fabrics. The loaded amount of Zn contents on the cotton fabric was determined by using induced coupled plasma atomic emission spectroscopy (ICP-AES). The surface morphological characterization of deposited ZnO NPs was examined, employing scanning electron microscopy (SEM), X-ray powder diffraction (XRD) and, Fourier- transform infrared spectroscopy (FTIR). The characterization results showed the presence of ZnO NPs on cotton fabrics having hexagonal wurtzite crystalline structure. The synthesized ZnO NPs on fabrics exhibited promising results for antibacterial, UV protection, and photo catalytic performance.

## 1. Introduction

Metal oxides have increased the interest in multiple applications for manufacturing due to their remarkable properties. The crystalline structure metal oxides are even more effective at nano scale due to their advanced mechanical, physical, and chemical properties. These specific properties of metal oxides in connection to the enhancement of surface area at the nanometer scale(less than 100 nm) [1,2,3,4]. In textile fabrication, metal oxides can be used to protect fabrics from UV, microbes, retard flame, conduct electricity, repel water, self-clean, etc. [5,6,7,8]. Zinc oxide (ZnO), titanium dioxide (TiO_2_), copper oxide (CuO), and magnesium oxide (MgO) are commonly used metal oxides in the textile industry as antibacterial agents [9,10,11,12]. For the UV protection and self-cleaning functionalities, nano-sized, and crystalline structure zinc oxide and titanium dioxide (TiO_2_) are mostly used [13,14]. These inorganic metal oxides in the form of nanostructure are more effective and stable than organic materials used for the same application [15]. The nanostructures of metallic oxides release ions, and these ions cause the inactivation of the bacteria protein through reaction with a thiol group (-SH) in protein, which eventually kill bacteria [16]. The metal oxide nanoparticles can scatter, absorb, and block the UV radiation; they stop the UV radiation and shield the skin from dire effects [15,17].

Zinc oxide is an inorganic metal oxide in the form of white powder. It is a versatile, non-toxic, and easy to synthesize multipurpose material having low-cost production, outstanding thermal, chemical, and mechanical stability [18]. ZnO is an n-type semiconductor material with a direct and wide band gap (3.37 eV), high excite on binding energy (60 meV), and high electron mobility [16,19,20,21]. The extraordinary photo catalytic properties due to the electronic structure of ZnO nanoparticles also empower them to be used as degradation reagents for various types of pollutants; e.g., it can be used in the textile industry for dyes and surfactants degradation in the waste water. ZnO is made of Zn^2+^ and O^2+^ ions that are bonded with each other with a considerable extent of ionicity and with a minor degree of covalency [16,22,23,24,25,26,27].

Up to date, a number of articles address the research on the production of ZnO nanoparticles [16].

There are mainly two approaches for production: the top-down approach (metallurgical process); which involves the heating of the precious mineral zincite [23], and the bottom-up approach; which incorporates chemical, mechanical, and physical processes. Other methods, such as the sol-gel process, sonochemical, solvochemical, microwave irradiation method, hydrothermal system, precipitation techniques, and microemulsion are also used [28,29,30,31,32].

The multiple parameters and different conditions involved during the synthesis process, for example, synthesis method, reagents used, pH of the medium, the temperature of the process, the processing time, concentrations of the precursors, reaction medium of the process, drying temperature, and time, all of these influence the amount produced, structure and size of ZnO nanoparticles, as well as their performance and functional properties [18,23,28,33]. The crystalline ZnO nanoparticles have mainly been found in three crystal structures, i.e., wurtzite hexagonal structure, cubic zinc blende, and cubic rocksalt structure. However hexagonal wurtzite structure is thermodynamically stable under typical conditions; therefore this structure is commonly available [34].

ZnO nanoparticles are deposited onto textile materials mainly by the ex-situ route. However, pre-synthesized ZnO nanoparticles show minor adsorption onto the textile materials. Moreover, the ex-situ route leads to a pile-up of nanoparticles on the surface of textile fiber, which results in the reduction of functional properties of textile materials [35,36,37]. In-situ growth of ZnO onto the fabric is an alternative to ex-situ deposition. It has been observed that in-situ deposition results in a homogeneous distribution of nanoparticles, moreover they demonstrate excellent adsorption and adhesiveness, thus imparting long-lasting functional properties [23].

One of the most common and advantageous processes for ZnO nanoparticle synthesis is a hydrothermal process. This process has attracted immense interest as one of the most favorable techniques for controlling particle size. Hong et al. reported on hydrothermal application for seedless growth of ZnO on different fabrics using [Zn(NO_3_)_2_·6H_2_O] as precursor (with nano rod structure) [38]. In another study, Baurah et al. adopted a hydrothermal process at 90 °C using [Zn(NO_3_)_2_·6H_2_O] as precursor to develop cotton fabric based composite material containing ZnO nano rods (with average particle size 81 nm) [39]. The hydrothermal method for ZnO has several advantages; moderate conditions are involved in the reaction, leading to nano-sized ZnO particles; with crystalline hexagonal wurtzite structure [40,41].

Shaheen et al. [42], El-Naggar et al. [43], and Zhang et al. [44] reported in-situ deposition of ZnO nanoparticles on textile fabrics; however, their approach in principle used a seeding method, where the textile fabric is immersed in a bath at a temperature range of 80 to 130 °C for the nuclei formation and further growth of nanoparticles on the fabric. The major disadvantage of the seeding method is its incompatibility on an industrial scale because of its time consuming approach [18,42,43,44].Therefore, there is a great need for an efficient method that is appropriate for the industrial scale of ZnO NPs on textile synthesis.

In this research work, the synthesis of ZnO nanoparticles on 100% cotton fabrics by one step in-situ hydrothermal method was investigated; UV protection, photocatalysis, and antibacterial properties were evaluated. Citric acid treatment for the surface activation leads to better adsorption and adhesion of ZnO nanoparticles to the 100% cotton fabrics. The optimal conditions to obtain the ZnO NP-based multifunctional textile including precursor ratios vs. the deposited amount of ZnO NPs on cotton are discussed.

## 2. Materials and Methods

### 2.1. Materials

The 100% cotton fabric with plain weave structure, 150 g/m^2^ aerial density, 27 ends/inch, 23 picks/inch were purchased from the Lincolor industry Czech Republic. Citric acid C_6_H_8_O_7_(CA), methyl orange (MO), zinc nitrate hexahydrate [Zn(NO_3_)_2_·6H_2_O], and sodium hydroxide (NaOH) precursors were purchased from Sigma- Aldrich, Prague, Czech Republic. All the chemicals were of analytical standard and used as obtained without any further purification.

### 2.2. Surface Activation of Cotton

For maximum adhesion of ZnO NPs onto the cellulose structure of cotton fabric, the cotton fabric was pretreated with a 0.5% aqueous solution of citric acid for surface activation. As the citric acid and cotton fibers are added in deionized water, both are ionized as shown in Equations (1) and (2). In further reactions, carboxylic groups of citric acid were easily attached to the hydroxyl groups on cotton fabric, as shown in Equation (3) [45].

(1) $$C_{6}H_{8}O_{7}+{\;H}_{2}O\;↔C_{6}H_{7}O_{7}^{-}+{\;H}_{3}O^{+},$$

(2) $$\mathrm{Cellulose}-\mathrm{OH}+{\;H}_{2}O\;↔\mathrm{Cellulose}-O+{\;H}_{3}O^{+},$$

(3) $$C_{6}H_{7}O_{7}^{-}+\mathrm{Cellulose}-\mathrm{OH}+H_{2}O\;↔\;\mathrm{Cellulose}-\mathrm{CA}+{\;H}_{3}O^{+}.$$

### 2.3. In-Situ Synthesis of Zinc Oxide on Cotton

**Figure 1:**
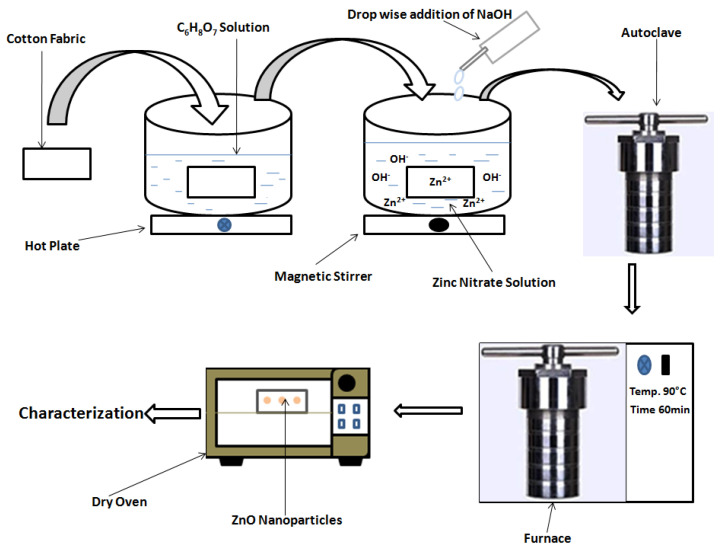
Schematic diagram of in-situ synthesis on ZnO nanoparticles onto the cotton fabrics.

ZnO nanoparticles were deposited on cotton fabric by the hydrothermal method. 0.1 M, 0.25 M, 0.5 M solutions of Zn(NO_3_)_2_·6H_2_O, and NaOH were prepared in deionized water. Then, 100 mL solution of NaOH was added drop-wise, vigorously stirred with 100 mL solution of Zn(NO_3_)_2_·6H_2_O. After 5 min of stirring 50 mL of the resulted solution was transferred into the autoclave. Then, the cotton fabric piece (5 cm × 5 cm) was put into the autoclave. Afterwards the autoclave was put into the furnace and heated at 90 °C for 1 h. After 1 h the autoclave was cooled at room temperature and the cotton fabric piece was taken out from the autoclave and dried in the oven at 90 °C for 2 h. The experimental design with different concentrations of Zn(NO_3_)_2_·6H_2_O and NaOH, as well as resulting responses of deposited amount of Zn contents onto fabric are shown in Table 1. Equations (4)–(7) describe the mechanism of ZnO nanoparticle synthesis on cotton fabric. A schematic diagram of ZnO nanoparticles loaded onto the cotton fabric is shown in Figure 1.

(4) $$\mathrm{Zn}{\left({\mathrm{NO}}_{3}\right)}_{2}\cdot 6H_{2}O\;\overset{H_{2}O}{\to }{\;\mathrm{Zn}}^{2+}+2{\mathrm{NO}}_{3}^{-}+6H_{2}O,$$

(5) $$\mathrm{NaOH}\;\overset{H_{2}O}{\to }{\;\mathrm{Na}}^{+}+{\;\mathrm{OH}}^{-},$$

(6) $${\mathrm{Zn}}^{2+}+4{\mathrm{OH}}^{-}\to \;\mathrm{Zn}{\left(\mathrm{OH}\right)}_{4}^{2-},$$

(7) $$\mathrm{Zn}{\left(\mathrm{OH}\right)}_{4}^{2-}\;\to \mathrm{ZnO}+H_{2}O+2{\mathrm{OH}}^{-}.$$

### 2.4. Characterization and Functional Properties

An induced coupled plasma atomic emission spectrometer (ICP AES, Optima 7300 DV, Perkin-Elmer Corporation, Waltham, MA, USA) was used to analyze the Zn contents. The developed samples of ZnO NPs loaded cotton fabric of weight 0.1 g were treated with 8 mL of concentrated HNO_3_ (65%) until the cotton completely dissolved. After that, obtained solutions were transferred to a 100 mL volumetric flask and then diluted with deionized water.

The surface of the ZnO coated textile was visualized employing Schottky type field emission gun scanning electron microscope (SEM) Quanta 200 FEG (FEI company, Hillsboro, OR, USA). Aiming to avoid charging effects of the insulating samples, they were investigated under a water vapor atmosphere in the low vacuum regime of 80 Pa utilizing a large field detector.

XRD patterns were recorded employing D8 Discover X-ray diffractometer (Bruker ASX GmbH, Billerica, MA, USA). Measurements were performed in the range 5.0–90.0° with a step size of 0.027° and auto-repeat function enabled. Diffractograms were processed with the DIFFRAC.EVA software. The size of nano crystallites was calculated by the Scherrer Equation (8) [46].

(8) $$d=\frac{K\lambda }{\beta \mathrm{Cos}\theta },$$

where K indicates the Scherrer constant (0.89), is the wavelength of X-rays, θ represents the Bragg diffraction angle, and β is full width at half maximum of the peak of ZnO nanoparticles.

Fourier Transform Infrared (FTIR) spectra were collected at room temperature using a “VERTEX 70” (Bruker, Ettlingen, Germany) spectrometer. Scans were made in attenuated total reflectance (ATR) mode using ZnSe crystal in a range from 4000 to 600 cm^−1^ with a resolution of 4 cm^−1^ and averaged over 16 scans.

The UV protection capability of obtained samples was judged using Varian CARY 1E UV/VIS spectrophotometer containing a DRA-CA-301 integrating sphere and solar screen software in the range 280–400 nm as per AATCC 183–2000 standard. According to this standard test method, the ultraviolet radiation transmitted or blocked by textile fabrics is determined for the samples intended to be used for UV protection. The percentage of UV radiation transmission was measured. Standard conditions of 20 ± 2 °C temperature and 65 ± 2% relative humidity were used for sample handling. The averaged value of four measurements was used. Equation (9) was used for UV protection factor (UPF) calculation:

(9) $$\mathrm{UPF}=\frac{\sum_{280\mathrm{nm}}^{400\mathrm{nm}}E_{\lambda }S_{\lambda }\Delta_{\lambda }}{\sum_{280\mathrm{nm}}^{400\mathrm{nm}}E_{\lambda }S_{\lambda }T_{\lambda }\Delta_{\lambda }},$$

where

E_λ_ = solar spectra irradiance
S_λ_ = relative erythemal spectral response
Δ_λ_ = measured wavelength region in nm
T_λ_ = average spectral transmittance (%)

Antibacterial performance of the fabrics was evaluated for Gram-negative *E. coli* and Gram-positive *S. aureus* bacteria organisms, as per quantitative test method AATCC 100–2012. This test method defines the process of obtaining the values for calculation of the decrease of bacteria colony amount, which appeared after 24-h exposure.

Samples with and without ZnO NPs were cut into pieces of 4.8 ± 0.1 cm diameter and stored in a 250 mL conical flask containing 1 mL of bacterial inoculums. After 24 h of the contact period, the solution was diluted in a nutrient bath. Further, the agar plates were prepared by putting the obtained solution into the agar plates. Then, the agar plates were incubated for 24 h at 37 ± 2 °C. The numbers of bacterial colonies that appeared in the agar plate were counted. The Equation (10) was used to calculate the bacterial reduction, expressed as a percentage.

(10) $$R\%=\frac{A-B}{A}\times 100,$$

where A is the amount of bacteria colonies recovered from the control sample; B is the amount of bacteria colonies from the treated cotton sample, and R% is the decrease of bacteria colonies in percent.

The photo catalytic ability of the ZnO NPs treated samples was examined based on the degradation of methyl orange under the influence of sunlight for 24 h. To perform the experiment, samples were placed in a 0.01% (weight/volume) solution of MO for 1 h in order to reach the equilibrium. Afterward, the samples were left to dry in the laboratory ambient. Finally, samples were exposed to sunlight illumination for different periods and the colour difference was assessed according to CIE Lab colour space using Equation (11) [47].

(11) $$\Delta E=\sqrt{{\left(\Delta L^{*}\right)}^{2}+{\left({\Delta a}^{*}\right)}^{2}+{\left({\Delta b}^{*}\right)}^{2}},$$

(12) $$\Delta L^{*}={\;L}_{1}^{*}-L_{2}^{*},$$

(13) $$\Delta a^{*}={\;a}_{1}^{*}-a_{2}^{*},$$

(14) $$\Delta b^{*}={\;b}_{1}^{*}-b_{2}^{*},$$

where ΔE is the colour change, L* lightness, a*, and b* are the chromaticity coordinates; which represent the colour directions: +a* is the red axis, −a* is the green axis, +b* is the yellow axis, and −b* is the blue axis.

## 3. Results

### 3.1. Induced Coupled Plasma Atomic Emission Spectroscopy (ICP-AES) Analysis

**Figure 2:**
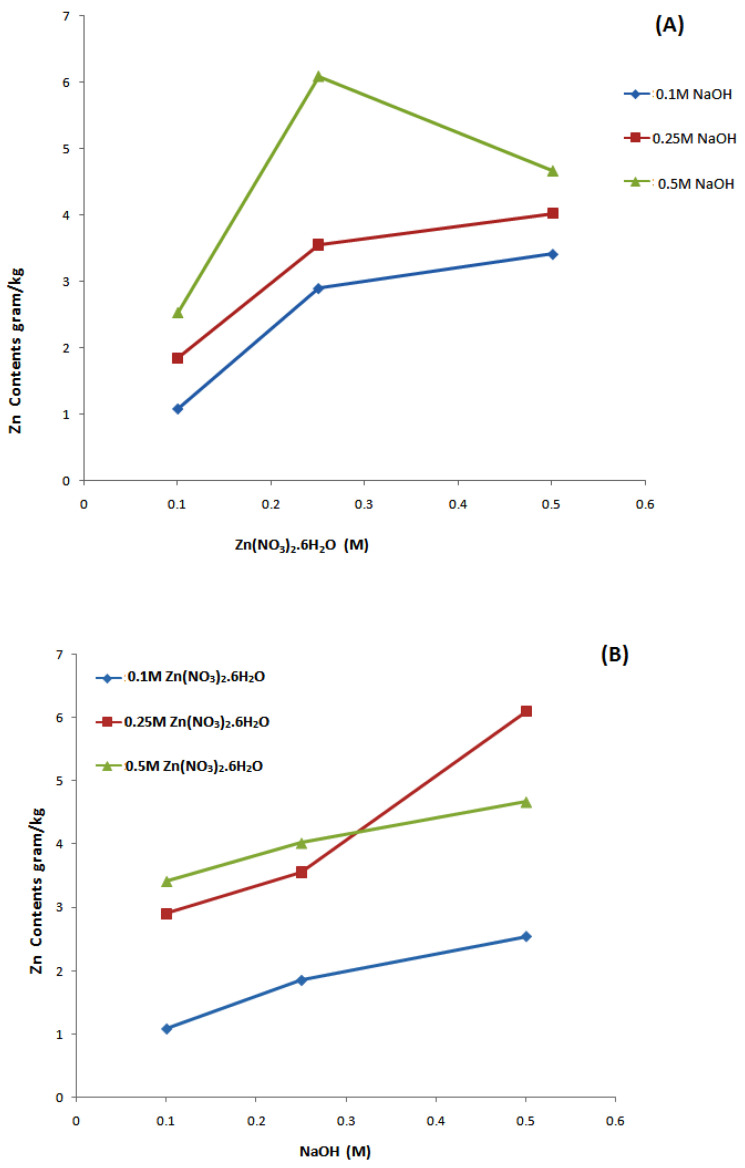
Deposited amount of Zn content versus precursor concentration: (**A**) Zn(NO_3_)_2_·6H_2_O, (**B**) NaOH.

Cotton fabric samples with deposited ZnO NPs were analyzed with the help of ICP-AES to determine the optimized and most effective molar concentrations of chemical precursors. From Table 1 and Figure 2 it can be seen that molar concentrations of chemical precursors have a great effect on the amount of Zn contents synthesized. The maximum amount of Zn contents was observed for sample 6, which showed Zn contents 6.091 g/kg. The optimal concentrations of precursors for these experiments are 0.25 M Zn(NO_3_)_2_·6H_2_O and 0.5 M NaOH. The calculated probability values (*p* values), as shown in Tables S1 and S2, Zn(NO_3_)_2_.6H_2_O = 0.00009 and NaOH = 0.00008 are less than 0.05, which proved the significant effect of precursors molar concentration on the amount of Zn contents synthesized, which indicates strong evidence against the null hypothesis. Moreover, Pearson correlation coefficients for Zn(NO_3_)_2_·6H_2_O = 0.572 and NaOH = 0.576 (Tables S1 and S2) indicates the positive correlation between molar concentration of precursors and the amount of Zn contents synthesized.

### 3.2. Surface Morphology

**Figure 3:**
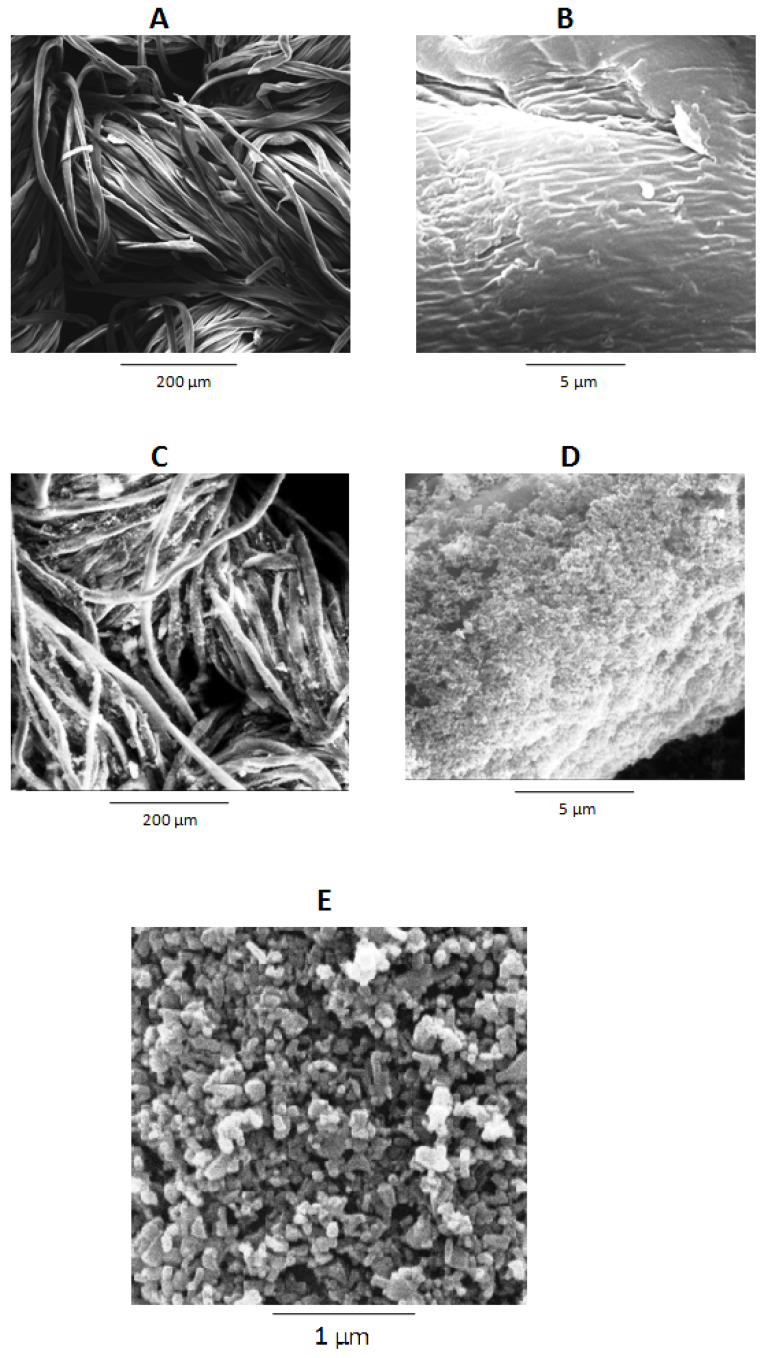
SEM images (**A**,**B**) pristine cotton. (**C**–**E**) highest amount of Zn contents, i.e., 6.091 g/kg (Sample 6).

The differences in the surface morphology of the pristine cotton sample and the ZnO NPs-loaded sample are seen in the SEM micrographs depicted in Figure 3 and Figure S1. The pristine cotton sample (Figure 3A,B) has some micro-roughness on the surface, which eventually increases the adhesion of the ZnO NPs during the chemical deposition. Conversely, the hydrothermal process treated cotton fabric (Figure 3C–E) becomes decorated with a ZnO NPs layer that was deposited with the optimized process No. 6 (Table 1). These ZnO NPs are distributed homogeneously on the surface of the sample 6. The presences of the ZnO NPs on the surface of sample 6 are due to the attraction between active functional groups of cellulose and ZnO NPs [48], which results in the cotton surface coverage by a layer of ZnO NPs.

### 3.3. XRD Analysis

**Figure 4:**
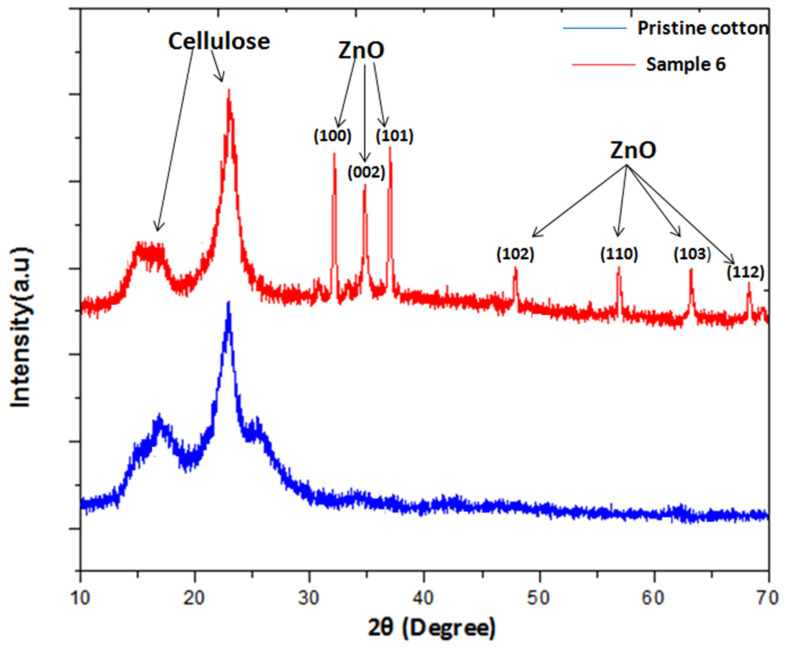
XRD diffractogram of pristine cotton and sample (6).

Figure 4 shows the X-ray diffraction pattern of pristine cotton and after one-step hydrothermal process treated sample 6. From the XRD it can be seen that pristine cotton has multiple peaks at 2θ = 14.9°, 16.6°, and 22.7°, which correspond to planes (1–10), (110), and (002), respectively. These are the characteristic diffraction peaks of cellulose (JCPDS 03-0226) [49]. On the other hand, the XRD pattern for ZnO NPs coated sample 6 shows that there are extra diffraction peaks at 2θ = 32.1°, 34.7°, 36.5°, 47.8°, 56.7°, 63.1°, and 68.1°. The characteristic peaks in XRD pattern polycrystalline hexagonal wurtzite (joint committee on the powder diffraction standard No. 36-1451) ZnO NPs were attributed to (1 0 0), (0 0 2), (1 0 1), (1 0 2), (1 1 0), (1 0 3), and (1 1 2) crystallographic plane orientations, respectively [50]. The crystallite size was calculated employing Equation (8) for (1 0 1) reflex. Samples with loaded ZnO NPs had crystallite sizes in the range 20.3–22.4 nm. Other studies report various crystallite sizes from 4 to 35 nm (Table 2).

### 3.4. FTIR Analysis

**Figure 5:**
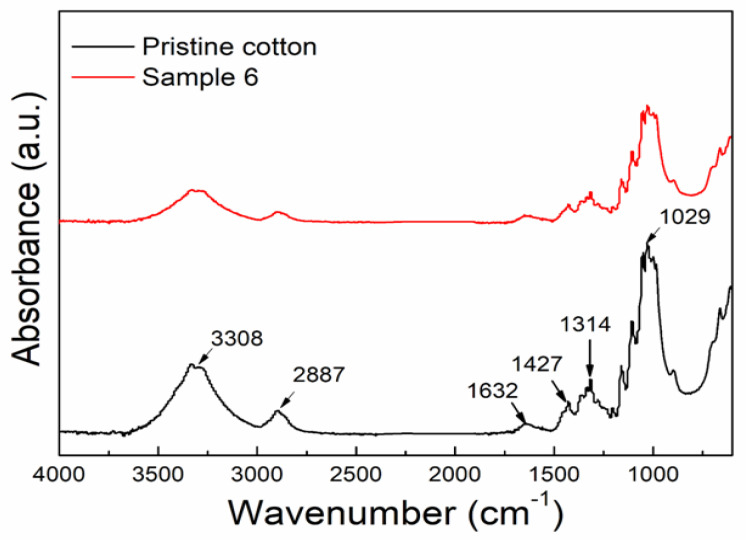
FTIR absorbance spectra of pristine cotton and sample (6).

The FTIR spectra were collected to examine the grafting information of in-situ deposited ZnO NPs on the surface of cotton fabric after a one-step hydrothermal process. The FTIR spectra of pristine cotton fabric and sample 6 are shown in Figure 5. The main detected bands at 3308 cm^−^^1^ (OH stretch) 2887 cm^−^^1^ (CH stretching), 1632 cm^−^^1^ (C=O stretching) [54], 1427 cm^−^^1^ (C-H wagging), 1314 cm^−^^1^ (C-H bending), and 1029 cm^−^^1^ (C-O stretch) are indicated in Figure 5 and all can be attributed to the cotton [55,56]. It can be seen from Figure 5 that after the one-step hydrothermal process the spectra of sample 6 have the same shape and characteristic peaks as one untreated cotton. The visible difference is the intensity of the peaks affirming that the cotton surface is covered by ZnO NPs [57,58].

### 3.5. UV Protection Factor

The spectrum of sunlight contains UV radiations, which have three main types: ultraviolet A, B, and C radiation (UVA, UVB, and UVC, respectively) [59]. The UVC covers the wavelengths from 100 to 280 nm, UVB from 280 to 315 nm, and UVA from 315 to 400 nm. UVC radiation is mostly absorbed by the ozone layer in the atmosphere and does not arrive on earth. The UVA radiation is the most harmful of all mentioned and causes skin cancer, sunburn, acne, and damages the DNA [60,61,62]. The ability of the fabric to provide skin shielding against harmful radiation is quantified as UV protection factor (UPF) [63]. The UPF value describes how much of the UV rays the fabric transmits. UPF values and protection categories of the fabric categorized by The Australian Standardization Institute is presented in Table S3 [64].

**Figure 6:**
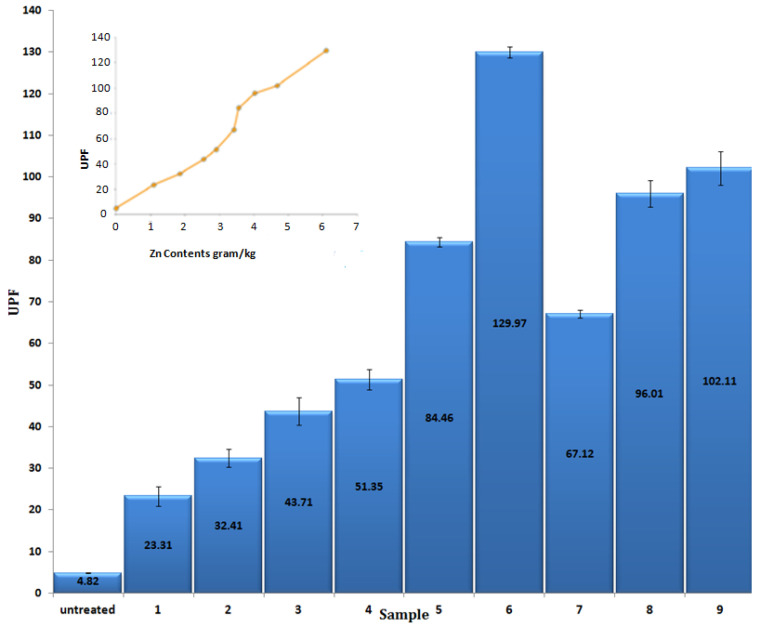
UPF values of untreated and ZnO nanoparticles loaded on cotton fabrics. The inset shows dependence of UPF values on the amount of Zn contents.

**Figure 7:**
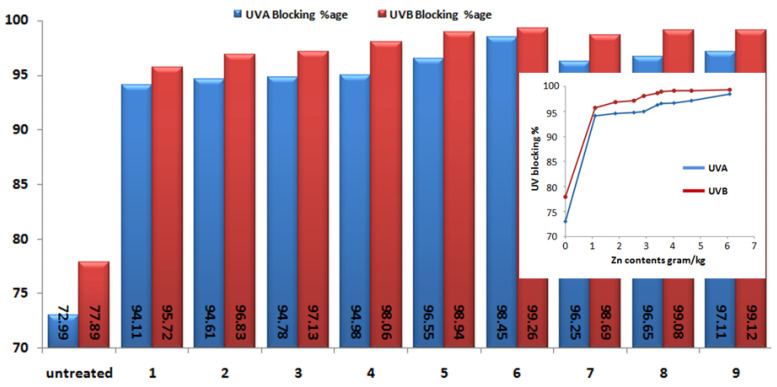
UVA and UVB blocking percentage of initial and cotton with ZnO NPs. The inset shows UV blocking percentage versus amount of Zn contents.

UV protective factor (Figure 6) and UVA and UVB blocking (Figure 7) of the one-step in-situ ZnO NPs loaded cotton is demonstrated. The initial cotton fabrics before loading had a value of only 4.82 UPF units, while the UVA and UVB blocking percentage values for the initial cotton fabrics were 72.99% and 77.89%, respectively. From the measured values it can be seen that after ZnO NPs loading UPF values as well as UVA and UVB blocking percentage increased, which can be explained by the UV absorption capacity of ZnO NPs on cotton fabric [16]. Furthermore, the highest UPF had a sample with highest Zn contents 6.091 g/kg (optimized sample 6), which was 129.97, which indicates that UPF values are strongly related to the amount of ZnO NPs content deposited onto the textile fabric. The Figure 6 inset indicates that UPF value increased with increased amount of Zn contents loaded on to the fabric. The sample with the highest Zn contents 6.091 g/kg (optimized sample 6) showed UVA and UVB blocking percentages as 97.11% and 99.12%respectively. Moreover Figure 7 inset shows that the UVA and UVB blocking percentages were increased with amount of Zn contents loaded on to the fabric. Calculated Pearsons correlation coefficient value (0.98) (Table S4) shows strong positive linear correlation between deposited amount of Zn contents and UPF value. Furthermore, the *p* value 0.0003 < 0.05 (Table S4) demonstrates that Zn contents amount influence the UPF value. UV blocking relation to the amount of Zn contents (Figure 7 inset) demonstrates that the increase of the deposited amount of Zn contents leads to both UVA blocking and UVB blocking percentage increase. The *p* values 2.6 × 10^−^^12^ < 0.05 and 5.6 × 10^−^^13^ < 0.05 (Tables S5 and S6) determine that the synthesized amount of Zn contents on to the fabric has a significant effect on UV blocking.

ZnO NPs highly absorb UV, moreover, the high refractive index of ZnO NPs has a great role in high UPF value and UV blocking. The high refraction ZnO NPs causes the scattering of UV light, therefore UV light is not transmitted to human skin [65]. Table 3 shows the comparisons of current results with literature.

### 3.6. Antibacterial Performance

**Figure 8:**
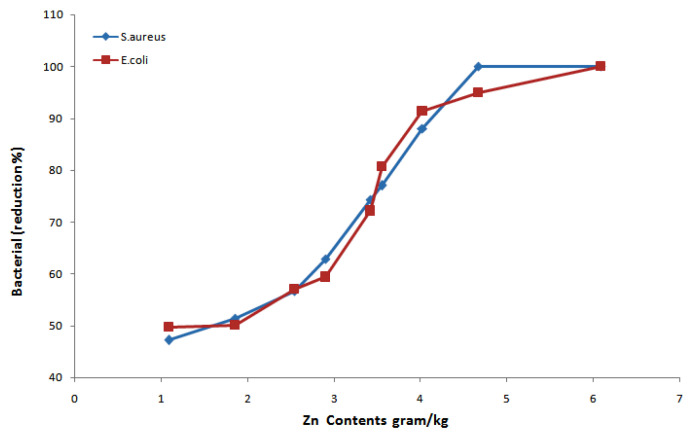
Bacterial reduction efficiency dependence on the Zn content.

Antibacterial performance of ZnO NPs loaded cotton fabrics was evaluated according to the colony count method (Table 4, Figure 8). The antibacterial activity of ZnO NPs loaded samples is increased with the increased amount of synthesized ZnO NPs on the cotton fabric for both Gram-negative *E. coli* and Gram-positive *S. aureus* bacteria. Sample 9 showed a 100% reduction only for *S. aureus* bacteria, while the optimized sample 6 showed a 100% bacterial reduction for both *E. coli* and *S. aureus* bacteria.

The antibacterial activity of ZnO NPs is due to the deactivation of vital enzymes caused by interacting with the thiol group and ZnO NPs, as well as being involved with the disruption of the bacteria membranes by ZnO NPs [17,56]. Moreover, as the ZnO NPs attach to the bacterial cell wall, it might result in an increased concentration of Zn^2+^ cations in the bacterial cytoplasm, which causes the death of bacterial cells [70]. Pearson correlation coefficient for *S. aureus* reduction and *E. coli* reduction percentage was calculated as 0.95 and 0.94 (Tables S7 and S8), respectively, which reflects the strong correlation with the amount of Zn contents deposited on the fabric surface. Furthermore, calculated *p*-values (Tables S7 and S8) 1.7 × 10^−^^6^ and 1.6 × 10^−^^6^ < 0.05 indicate the significant effect of Zn amount on the bacterial reduction for both *S. aureus* and *E. coli.*

### 3.7. Photo Catalytic Ability

**Figure 9:**
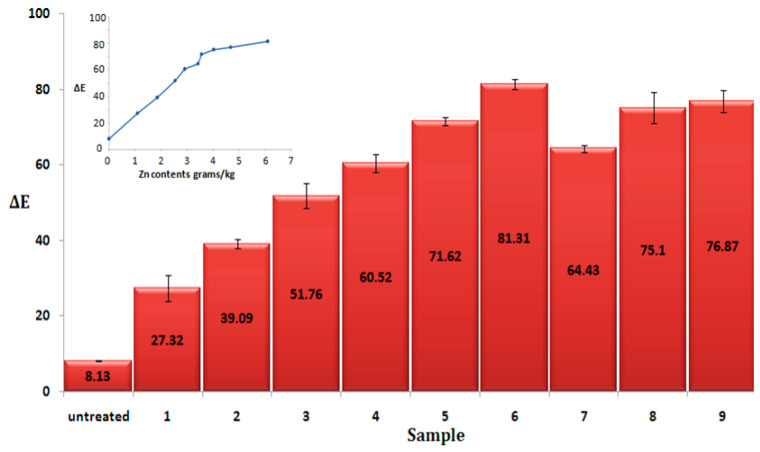
Photocatalytic properties (colour difference ∆E) of untreated and ZnO nanoparticles loaded cotton fabrics. The inse t shows colour difference (∆E) dependence on the Zn content. The line is for eye guidance.

**Figure 10:**
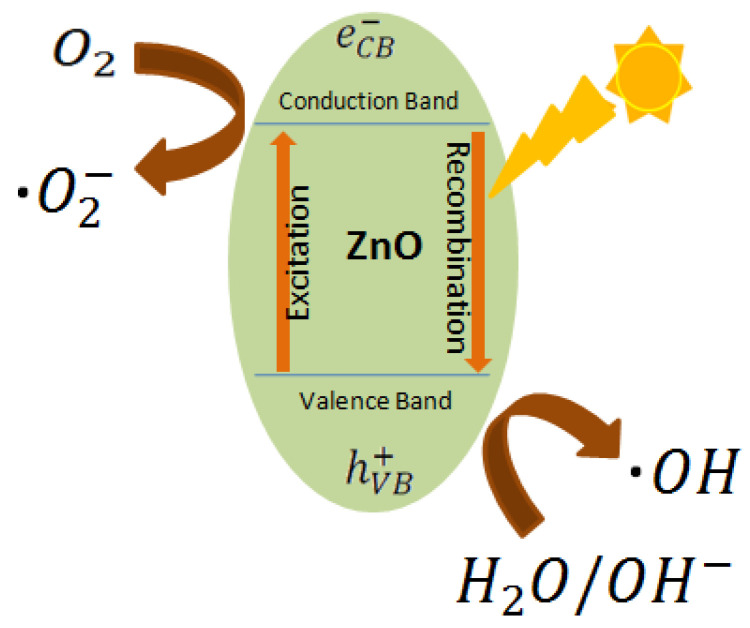
Schematic mechanism of photocytalytic activity.

The photo catalytic ability of ZnO NPs loaded cotton fabrics was assessed according to the measurement of the colour change (∆E) of methyl orange before and after exposure to sunlight illumination. Figure 9 shows the change in colour of the initial fabrics and ZnO NPs loaded cotton fabric samples and the schematic mechanism of photo catalytic activity in the Figure 10 [16,71]. It can be noticed that the initial fabric sample has little colour change (∆E), while on the other hand, the ZnO loaded samples demonstrated high photo catalytic activity. The photo catalytic performance of ZnO NPs loaded cotton fabric samples increased with the increased amount of ZnO NPs. In another study, Javed et al. reported the colour difference (∆E) 86.21 for ZnO NPs loaded cotton fabrics using a sonochemical method [56].

The Figure 9 inset shows the maximum colour difference (∆E) 81.31 observed for sample 6, having the maximum amount of ZnO NPs. These results are in line with the work by Sudrajat. H., demonstrating that the higher photo catalytic activity is obtained at higher amounts of ZnO NPs [72]. The calculated Pearson correlation coefficient is 0.95 (Table S9), showing that ∆E value has strong correlation with the amount of Zn contents synthesized. Furthermore, calculated *p* value 0.9 × 10^−^^5^ < 0.05 (Table S9) prove that the amount of Zn contents synthesized on to the fabric surface have a significant effect.

As compared to untreated cotton fabric, the higher photo catalytic activity of ZnO NPs loaded cotton fabrics is because of ZnO NPs on the surface of the cotton fabrics, since ZnO NPs cause degradation of the dye molecules by photo catalytic reactions [73]. In the photo-catalytic mechanism by ZnO NPs, two photochemical reactions “oxidation and reduction” occur. During these reactions reactive oxygen species “•OH and •O^2−^” are produced. The •OH radical has a vital role in oxidating the dye molecules [16]. The mechanism of dye degradation can be described by Equations (15)–(18).

(15) $$ZnO+hv\to ZnO\;\left(2h^{+}+2e^{\_}\right)$$

(16) $$\frac{1}{2}O_{2}+2e^{\_}\to H_{2}O+2OH^{-}$$

(17) $$2OH^{-}\;+h^{+}\to 2•OH$$

(18) •OH+dye→degradation

## 4. Conclusions

In this work, modification of cotton textile was shown by the in-situ one-step hydrothermal synthesis of zinc oxide nanoparticles on pre-treated 100% cotton fabric. XRD analysis revealed pure wurtzite hexagonal crystals, with nano crystallite size of ZnO NPs between 5–6 nm, indicating the crucial role of the hydrothermal process at low temperature, i.e., 90 °C. SEM, ICP AES, and FTIR indicated the presence and grafting of ZnO NPs on the cotton fabrics. Varying the molar concentrations of the precursors’ optimal conditions at 0.25 M of Zn(NO_3_)_2_·6H_2_O and 0.5 M of NaOH yielded 6.091 g/kg of Zn deposited on the fabric. The study revealed that the amount of ZnO NPs synthesized onto the cotton fabric directly correlated with the performance of functional properties. Antibacterial efficacy of 100% for *E. coli* and *S. aureus* bacteria was demonstrated. A UV protection factor reaching 130 for the sample containing the highest amount of Zn contents was demonstrated. Similarly, photo catalytic activity investigated using methyl orange confirmed the direct dependence of the colour change on the ZnO concentration. Our work demonstrates a feasible method for the high yield industrial loading of ZnO NPs on functional textile.

## Figures and Tables

**Table 1:** Molar concentrations of the reagents and resulting Zn contents.

Sample No.	Zn(NO_3_)_2_·6H_2_O (M)	NaOH (M)	Deposited Amount of Zn Contents onto Fabric (gram/kg)	Standard Deviation
Untreated	-	-	-	
1	0.1	0.1	1.089	0.041
2	0.1	0.25	1.851	0.034
3	0.1	0.5	2.541	0.061
4	0.25	0.1	2.902	0.052
5	0.25	0.25	3.553	0.071
6	0.25	0.5	6.091	0.095
7	0.5	0.1	3.418	0.064
8	0.5	0.25	4.021	0.066
9	0.5	0.5	4.671	0.083

**Table 2:** Comparison of crystallite sizes of ZnO NPs.

Method	Precursors	Crystallite Size in nm (XRD)	Ref.
One Step Hydrothermal	Zn(NO_3_)_2_·6H_2_O, NaOH	20.3–22.4	This study
Solvothermal	Zn(NO_3_)_2_·6H_2_O, tetramethylammonium hydroxide	4.1	[51]
Precipitation Method	Zinc acetate, Diethylene glycol	5–35	[52]
Solvothermal	Zinc acetate, Triethanolamine	33±2	[53]

**Table 3:** Comparison of UPF, UVA, and UVB blocking percentage.

Precursors	Size of ZnO NPs	Method	UPF	UVA Blocking (%)	UVB Blocking (%)	Mass Load Zn (g/kg)	Ref.
Zn(NO_3_)_2_·6H_2_O, NaOH	20.3–22.4 (by XRD)	One step hydrothermal	129.97	97.11	99.12	6.091	This work
ZnCl_2,_NaOH	-	One step hydrothermal	80.2	92.6	99.1	0.71	[18]
Zn(NO_3_)_2_·6H_2_O, C_6_H_12_N_4_	1760 ± 120 (by SEM)	Two step hydrothermal	157.8	99.54	99.69	-	[48]
Zn(NO_3_)_2_·6H_2_O, Hexamethyleneteraamine	55 ± 6.1 (by SEM)	Two step hydrothermal	114	95.8	99.3	-	[66]

**Table 4:** Antibacterial performance of ZnO nanoparticles loaded cotton fabrics.

Sample No.	Zn Contents (g/kg)	*S. aureus* (Reduction %)	*E. coli* (Reduction %)	Ref.
1	1.089	47.23	49.76	
2	1.851	51.41	50.12	
3	2.541	56.67	57.03	
4	2.902	62.85	59.43	This study
5	3.553	77.06	80.65	
6	6.091	100.00	100.00	
7	3.418	74.31	72.17	
8	4.021	88.09	91.47	
9	4.671	100.00	94.96	
Wet chemical method	-	>99.99	80	[67]
Solochemical process	-	100	-	[68]
Ultrasonic Irradiation Process	-	100	100	[69]

## Data Availability

Data is available on the request to the corresponding author.

## Supplementary Materials

The following are available online at https://www.mdpi.com/article/10.3390/ma14143956/s1, Figure S1: SEM Images A and C Zn contents 4.671 gram/kg (Sample 9). B and D Zn contents 4.021 gram/kg (sample 8), Table S1: Statistical analysis by *t*-test (effect of Zn(NO_3_)_2_·6H_2_O (M) on deposited amount of Zn Contents gram/kg), Table S2: statistical analyses by *t*-test (effect of NaOH (M) on deposited amount of Zn Contents gram/kg), Table S3: UPF value and protection category of the fabric categorized by The Australian standardization Institute, Table S4: Statistical analysis by *t*-test (effect of Zn Contents gram/kg on UPF value), Table S5: Statistical analysis by *t*-test (effect of Zn Contents gram/kg on UVA Blocking%), Table S6: Statistical analysis by *t*-test (effect of Zn Contents gram/kg on UVB Blocking%), Table S7: Statistical analysis by *t*-test (effect of Zn Contents gram/kg on *S. aureus* (Reduction%), Table S8: Statistical analysis by *t*-test (effect of Zn Contents gram/kg on *E. coli* (Reduction%), Table S9: Statistical analysis by *t*-test (effect of Zn Contents gram/kg on ∆E).
