# Supplementary material for: One Step In-Situ Synthesis of Zinc Oxide Nanoparticles for Multifunctional Cotton Fabrics
Source: Materials (Basel). 2021 Jul 15;14(14):3956. doi: 10.3390/ma14143956 (PMC8304503; doi:10.3390/ma14143956)
Supplement: Supplementary file 1 [file materials-14-03956-s001.zip › materials-1237203-supplementary.pdf]

materials

Supplementary Materials

One step *in-situ* synthesis of zinc oxide nanoparticles for multifunctional cotton fabrics

Asif Javed[1*], Jakub Wiener[1], Asta Tamulevičienė[2,3], Tomas Tamulevičius[2,3], Algirdas Lazauskas[2], Jana Saskova[1], and Simas Rackauskas[2,3]

[1] Department of Material Engineering, Faculty of Textile Engineering, Technical University of Liberec, Liberec 46001, Czech Republic
[2] Institute of Materials Science, Kaunas University of Technology. K, Baršausko St. 59LT-51423 Kaunas, Lithuania
[3] Department of Physics, Kaunas University of Technology, Studentų St. 50, Kaunas LT-51423, Lithuania
* Corresponding author e-mail: asif.javed@tul.cz

Table S1 Statistical analysis by t –test (effect of $Zn(NO_3)_2.6H_2O$ (M) on deposited amount of Zn Contents gram/kg)

	$Zn(NO_3)_2.6H_2O$ (M)	Zn contents(gram/kg)
Mean	0.283	3.349
Variance	0.031	2.245
Observations	9	9
Pearson Correlation	0.572	
Hypothesizes Mean Difference	0	
df	8	
t stat	-6.5416	
P(T<=t) one tail	0.00009	
t critical one tail	1.8595	
P(T<=t) two tail	0.00018	
t critical two tail	2.306	

Table S2 statistical analyses by t –test (effect of NaOH (M) on deposited amount of Zn Contents gram/kg)

	NaOH (M)	Zn contents(gram/kg)
Mean	0.283	3.349
Variance	0.031	2.245
Observations	9	9
Pearson Correlation	0.576	
Hypothesizes Mean Difference	0	
df	8	
t stat	-6.5453	
P(T<=t) one tail	0.00008	
t critical one tail	1.8595	
P(T<=t) two tail	0.00017	
t critical two tail	2.306	

Table S3 UPF value and protection category of the fabric categorized by The Australian standardization Institute[1]

UPF Value	Protection Level
Below 15	Not good
15-24	Good
24-39	Very good
40 and above	Excellent

Table S4 Statistical analysis by t –test (effect of Zn Contents gram/kg on UPF value)

	Zn contents(gram/kg)	UPF
Mean	3.01	63.529
Variance	3.11	1553.63
Observations	10	10
Pearson Correlation	0.98	
Hypothesizes Mean Difference	0	
df	9	
t stat	-5.078	
P(T<=t) one tail	0.00033	
t critical one tail	1.833	
P(T<=t) two tail	0.00066	
t critical two tail	2.262	

Table S5 Statistical analysis by t –test (effect of Zn Contents gram/kg on UVA Blocking%)

	Zn contents(gram/kg)	UVA Blocking%
Mean	3.01	93.64
Variance	3.11	54.46
Observations	10	10
Pearson Correlation	0.73	
Hypothesizes Mean Difference	0	
df	9	
t stat	-46.17	
P(T<=t) one tail	2.6×10^{-12}	

t critical one tail	1.833
P(T<=t) two tail	$5.2x10^{-12}$
t critical two tail	2.262

Table S6 Statistical analysis by t –test (effect of Zn Contents gram/kg on UVB Blocking%)

	Zn contents(gram/kg)	UVB Blocking%
Mean	3.01	96.07
Variance	3.11	42.21
Observations	10	10
Pearson Correlation	0.72	
Hypothesizes Mean Difference	0	
df	9	
t stat	-54.82	
P(T<=t) one tail	$5.6x10^{-13}$	
t critical one tail	1.833	
P(T<=t) two tail	$1.1x10^{-12}$	
t critical two tail	2.262	

Table S7 Statistical analysis by t –test (effect of Zn Contents gram/kg on *S.aureus*(Reduction%)

	Zn contents(gram/kg)	*S.aureus*(Reduction%)
Mean	3.348	73.06
Variance	2.245	400.47
Observations	9	9
Pearson Correlation	0.95	
Hypothesizes Mean Difference	0	
df	8	
t stat	-11.25	
P(T<=t) one tail	$1.7x10^{-6}$	
t critical one tail	1.86	
P(T<=t) two tail	$3.4x10^{-6}$	
t critical two tail	2.31	

Table S8 Statistical analysis by t –test (effect of Zn Contents gram/kg on *E.coli* (Reduction%)

	Zn contents(gram/kg)	*E.coli*(Reduction%)
Mean	3.348	72.84
Variance	2.245	389.26
Observations	9	9
Pearson Correlation	0.94	
Hypothesizes Mean Difference	0	
df	8	
t stat	-11.37	
P(T<=t) one tail	$1.6x10^{-6}$	
t critical one tail	1.86	

P(T<=t) two tail	$3.4x10^{-6}$
t critical two tail	2.31

Table S9 Statistical analysis by t –test (effect of Zn Contents gram/kg on ΔE)

	Zn contents(gram/kg)	ΔE
Mean	3.01	55.61
Variance	3.11	576.99
Observations	10	10
Pearson Correlation	0.95	
Hypothesizes Mean Difference	0	
df	9	
t stat	-7.44	
P(T<=t) one tail	$1.9x10^{-5}$	
t critical one tail	1.833	
P(T<=t) two tail	$3.9x10^{-5}$	
t critical two tail	2.26	

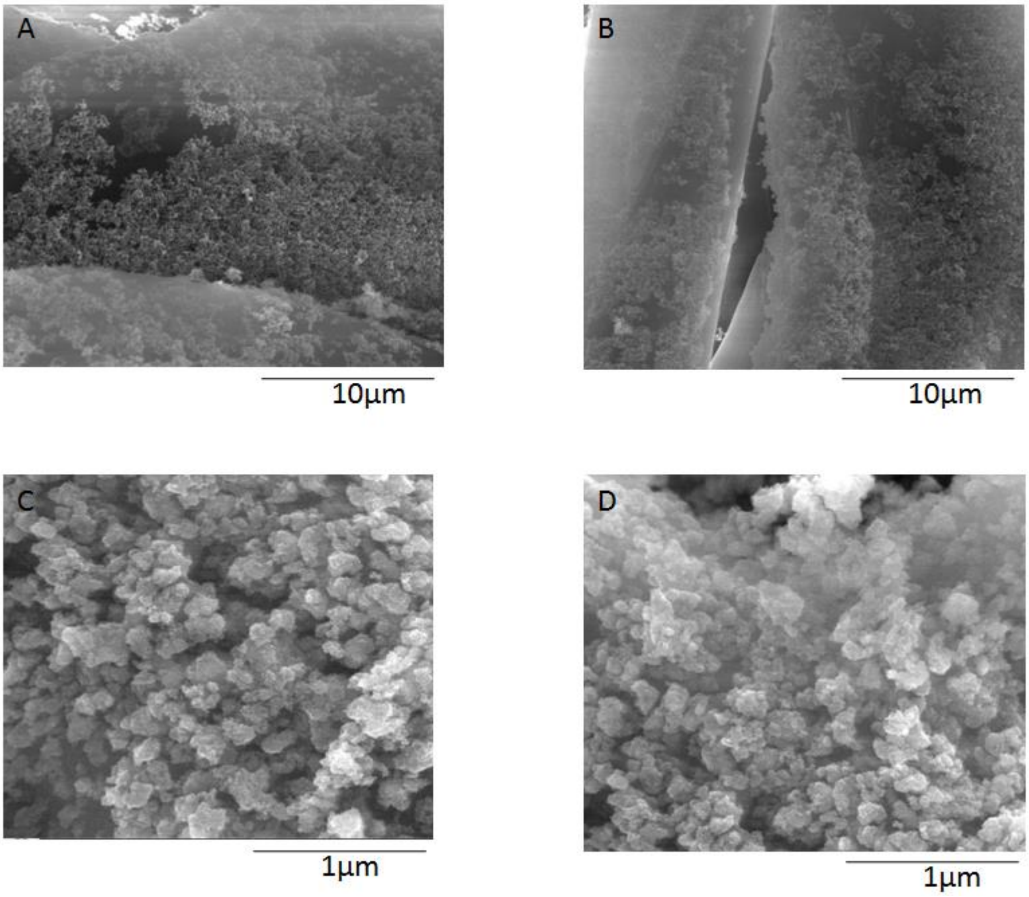

Figure S1 SEM Images A and C Zn contents 4.671 gram/kg (Sample9). B and D Zn contents 4.021 gram/kg (sample 8)

References

1. Gies, P.; Slevin, T.; Harrison, S.; Plowman, P.; Dain, S.; Moller, L.; Mawley, F.; Swift, N. *Australian/New Zealand Standard, AS/NZS 4399: 2017: Sun Protective Clothing–Evaluation and Classification*; Standards Australia, 2017; ISBN 1760358843.
